# Resolvin E1 Inhibits Substance P-Induced Potentiation of TRPV1 in Primary Sensory Neurons

**DOI:** 10.1155/2016/5259321

**Published:** 2016-09-25

**Authors:** Youn Yi Jo, Ji Yeon Lee, Chul-Kyu Park

**Affiliations:** ^1^Department of Anesthesiology and Pain Medicine, Gachon University, Gil Medical Center, Incheon 21565, Republic of Korea; ^2^Department of Physiology, College of Medicine, Gachon University, Incheon 21999, Republic of Korea

## Abstract

The neuropeptide substance P (SP) is expressed in primary sensory neurons and is commonly regarded as a “pain” neurotransmitter. Upon peripheral inflammation, SP activates the neurokinin-1 (NK-1) receptor and potentiates activity of transient receptor potential vanilloid subtype 1 (TRPV1), which is coexpressed by nociceptive neurons. Therefore, SP functions as an important neurotransmitter involved in the hypersensitization of inflammatory pain. Resolvin E1 (RvE1), derived from omega-3 polyunsaturated fatty acids, inhibits TRPV1 activity via activation of the chemerin 23 receptor (ChemR23)—an RvE1 receptor located in dorsal root ganglion neurons—and therefore exerts an inhibitory effect on inflammatory pain. We demonstrate here that RvE1 regulates the SP-induced potentiation of TRPV1 via G-protein coupled receptor (GPCR) on peripheral nociceptive neurons. SP-induced potentiation of TRPV1 inhibited by RvE1 was blocked by the G*α*i-coupled GPCR inhibitor pertussis toxin and the G-protein inhibitor GDP*β*-S. These results indicate that a low concentration of RvE1 strongly inhibits the potentiation of TRPV1, induced by the SP-mediated activation of NK-1, via a GPCR signaling pathway activated by ChemR23 in nociceptive neurons. RvE1 might represent a new therapeutic target for the treatment of inflammatory pain as a prospective endogenous inhibitor that strongly inhibits TRPV1 activity associated with peripheral inflammation.

## 1. Introduction

Large numbers of inflammatory mediators produced by various cells reacting to tissue damage are involved in regulating pain sensation [1, 2]. These secreted inflammatory substances act on specific receptors expressed by nociceptive sensory neurons resulting in the production of second messengers and the downstream activation of protein kinases and phospholipases. Second messengers subsequently trigger peripheral sensitization by controlling numerous receptors and ion channels [3, 4].

Peripheral substance P (SP) is a neurotransmitter of small-diameter nociceptive afferents in the somatic nervous system and in synaptic terminals of the spinal dorsal horn [5, 6]. Biological activities of SP are mediated by the activities of three different neurokinin (NK) receptors: NK-1, NK-2, and NK-3 [7]. The NK-1 receptor in particular has a strong affinity for SP [8]. Indeed, several studies support the idea that the SP-induced activation of NK-1 receptors in the processing of noxious information within the spinal cord is involved in inflammation and neuropathic pain [9–11].

Transient receptor potential vanilloid subtype 1 (TPRV1) is also specifically expressed by the nociceptors of C fibers and has an important role in triggering hyperalgesia by reacting to capsaicin, noxious heat, photons, and various endogenous ligands [12]. TRPV1 activity is enhanced by inflammatory mediators such as nerve growth factor, somatostatin, bradykinin, prostaglandins, and serotonin, suggesting that TRPV1 is essential for the integration of various signaling pathways that mediate sensitivity to stimuli [13]. SP is involved in the hypersensitization of inflammatory pain by activating NK-1 expressed by nociceptive neurons and potentiating the activity of TRPV1 upon peripheral inflammation. This suggests that the activation of NK-1 receptors by peripheral SP increases TRPV1 sensitivity and triggers hyperalgesia [14].

Resolvins are endogenous lipid mediators produced from *ω*-3 polyunsaturated fatty acids during resolution phase of acute inflammation that have strong resolving and anti-inflammatory effects [15, 16]. Resolvin E1 (RvE1) is derived from eicosapentaenoic acid. Low dose administration of RvE1 reduced inflammatory pain via the peripheral, spinal, and systemic management/inhibition of the inflammatory response and TRPV1 activity [15, 16]. Strikingly, RvE1 potently inhibited capsaicin-induced TRPV1 currents (IC_50_ = 1 nM) in dissociated dorsal root ganglion neurons. This IC_50_ was approximately 160 times lower than that of AMG9810, a commonly used TRPV1 antagonist [17, 18]. RvE1 inhibits TRPV1 activity via the G-protein coupled receptor chemerin 23 receptor (ChemR23). The RvE1 receptor expressed by primary sensory neurons induces an antinociceptive effect [16, 18]. These results indicate that ChemR23 is coexpressed by nociceptive neurons expressing NK-1 and TRPV1. Furthermore, low concentrations of RvE1 completely inhibited SP-induced TRPV1 potentiation via a G-protein coupled receptor (GPCR) signaling pathway activated by ChemR23. Taken together, we propose that RvE1 might function as an endogenous inhibitor of TRPV1 activity associated with peripheral inflammation.

## 2. Materials and Methods

### 2.1. Animals

All surgical and experimental procedures were reviewed and approved by the Institutional Animal Care and Use Committee of the College of Medicine at Gachon University. Adult C57BL/6 mice (male, 4–6 weeks) were purchased from Orientbio (Seongnam, Korea). Thirty mice were habituated for at least 1 week prior to experiments in a conventional facility with a 12:12 h light-dark cycle (lights on 8:00 am) and had* ad libitum* access to food and water.

### 2.2. Preparation of Dorsal Root Ganglion (DRG) Neurons

DRG neuron cultures were prepared as previously reported [17]. DRGs were aseptically removed from mice and incubated with collagenase (1.25 mg/mL, Roche, Indianapolis, IN)/dispase-II (2.4 units/mL, Roche) at 37°C for 90 min and then digested with 0.25% trypsin for 8 min at 37°C, followed by 0.25% trypsin inhibitor for 2 min at 37°C. Cells were mechanically dissociated with a flame polished Pasteur pipette in the presence of 0.05% DNAse I (Sigma, St. Louis, MO). DRG cells were plated on glass cover slips that were previously coated with a solution of 0.1 mg/mL poly-L-ornithine and grown in a neurobasal-defined medium (with 2% B27 supplement, Invitrogen) with 5 *μ*M AraC and 5% CO_2_ at 36.5°C. DRG neurons were grown for 24 h prior to further experimentation.

### 2.3. Single-Cell Reverse-Transcription Polymerase Chain Reaction (RT-PCR)

Single-cell RT-PCR was performed as previously described [19]. Briefly, a single cell was aspirated into a patch pipette with a tip diameter of about 25 *μ*m, gently placed into a reaction tube containing reverse-transcription reagents and incubated for 1 h at 50°C (Superscript III, Invitrogen, Carlsbad, CA). The cDNA product was utilized in a separate PCR. All primer sequences used for single-cell PCR are presented in Table 1. The first round of PCR was performed in 50 *μ*L of PCR buffer containing 0.2 mM dNTPs, 0.2 *μ*M “outer” primers, 5 *μ*L RT product, and 0.2 *μ*L platinum Taq DNA polymerase (Invitrogen). The protocol included a 5 min initial denaturation step at 95°C followed by 40 cycles of 40 s denaturation at 95°C, 40 s annealing at 55°C, and 40 s elongation at 72°C. The reaction was completed with 7 min of final elongation. For the second round of amplification, the reaction buffer (20 *μ*L) contained 0.2 mM dNTPs, 0.2 *μ*M “inner” primers, 5 *μ*L of the first-round PCR products, and 0.1 *μ*L platinum Taq DNA polymerase. The reaction procedure for these primers was the same as that used during the first round. A negative control was obtained from pipettes that did not harvest any cell contents yet were submerged in the bath solution. The PCR products were displayed on ethidium bromide-stained 2% agarose gels.

### 2.4. Whole-Cell Patch Clamp Recordings

Whole-cell voltage- and current-clamp recordings were performed at 24–28°C to measure currents and action potentials, respectively, using an Axopatch-200B amplifier (Axon Instruments, Union City). The patch pipettes were pulled from borosilicate capillaries (Chase Scientific Glass Inc., Rockwood, CA). When filled with the pipette solution, the resistance of the pipettes was 4-5 MΩ. The recording chamber (volume 300 *μ*L) was continuously superfused (2-3 mL/min). Series resistance was compensated for (>80%), and leak subtraction was performed. Data were low-pass-filtered at 2 KHz and sampled at 10 KHz. pClamp8 (Axon Instruments) software was used during experiments and analysis. The pipette solution for voltage-clamp experiments was composed of (in mM) 126 K-gluconate, 10 NaCl, 1 MgCl_2_, 10 EGTA, 2 NaATP, and 0.1 MgGTP, adjusted to pH 7.4 with KOH, with an osmolarity of 295–300 mOsm. In some cases, GDP*β*-S (2.5 mM) was included in the intracellular solution to block GPCRs. The extracellular solution for voltage-clamp experiments contained (in mM) 140 NaCl, 5 KCl, 2 CaCl_2_, 1 MgCl_2_, 10 HEPES, and 10 glucose, adjusted to pH 7.4 with NaOH, with an osmolarity of 300–310 mOsm. The extracellular solution was rendered Ca^2+^-free by adding 0 mM CaCl_2_ and 2 mM EGTA for the chelation of ambient Ca^2+^. Voltage-clamp experiments were performed at a holding potential of −60 mV. The pipette solution for current-clamp experiments was composed of (in mM) 145 K-gluconate, 2 MgCl_2_, 1 CaCl_2_, 10 EGTA, 5 HEPES, and 5 K_2_ATP, adjusted to pH 7.3–7.4 with KOH, with an osmolarity of 300 mOsm. The extracellular solution for current-clamp experiments contained (in mM) 140 NaCl, 5 KCl, 2 CaCl_2_, 1 MgCl_2_, 10 HEPES, and 10 glucose, adjusted to pH 7.4 with NaOH, with an osmolarity of 300–310 mOsm.

### 2.5. Ca^2+^ Imaging

Fura-2AM- (Molecular Probes, Eugene, OR) based Ca^2+^ imaging experiments were performed as previously described [19]. Briefly, cells loaded with fura-2AM (5 *μ*M) at 37°C were placed onto an inverted microscope (IX70, Olympus) and illuminated with a 175 W xenon arc lamp. Excitation wavelengths (340/380 nm) were selected using a Lambda DG-4 monochromatic wavelength changer (Sutter Instrument, Novato, CA). Intracellular free Ca^2+^ concentration ([Ca^2+^]i) was measured at 36°C via digital video microfluorometry using an intensified charge-coupled device camera (Cascade, Roper Scientific, Trenton, NJ) coupled to a microscope and a Pentium 5 computer with imaging software (Metamorphor, Universal Imaging Corp., PA).

### 2.6. Drugs

Capsaicin and SP (Sigma, St. Louis, MO) stock solutions were made in ethanol and H_2_O, respectively, and stored at −20°C. The drugs were diluted to their final concentration with the extracellular solution and then applied by gravity through a bath perfusion system. Guanosine 5′-[b-thio]diphosphate trilithium salt (GDP*β*-S) was obtained from Sigma. Pertussis toxin was purchased from Tocris Bioscience (Bristol, UK). Resolvins were initially isolated in exudates formed in the resolution phase of self-limited acute inflammation. After the full structural elucidation, resolvins were produced by full organic chemical synthesis [15, 20]. Synthetic RvE1 (5S,12R,18R-trihydroxy-6Z,8E,10E,14Z,16E-eicosapentaenoic acid) was obtained from Cayman Chemical (Ann Arbor, MI, USA) and was qualified according to published physical and biological properties [15, 21]. The stock solution contained 10 or 100 ng/*μ*L resolvin suspended in 100% ethanol and was kept in a −80°C freezer. Caution was taken to avoid the exposure of RvE1 to air during sample preparation. Immediately before use, RvE1 was directly diluted to test concentrations with the bath solution and briefly sonicated. The final concentration of RvE1 used was 0.5–3 nM based on our previous study [18]. The final ethanol concentration was always kept under 1%. Under such manipulations, no degradation of RvE1 was observed in mass/mass spectroscopy measurements (data not shown).

### 2.7. Statistical Analysis

All data were expressed as mean ± standard error of the mean (SEM). One-way analysis of variance (ANOVA) or unpaired Student's* t*-test was used to determine the differences using Origin 6.0 (Microcal Software, Inc., Northampton, MA). Differences were considered to be significant when *P* < 0.05.

## 3. Results

### 3.1. Coexpression of TRPV1, ChemR23, and NK-1 in Small-Sized DRG Neurons

**Figure 1:**
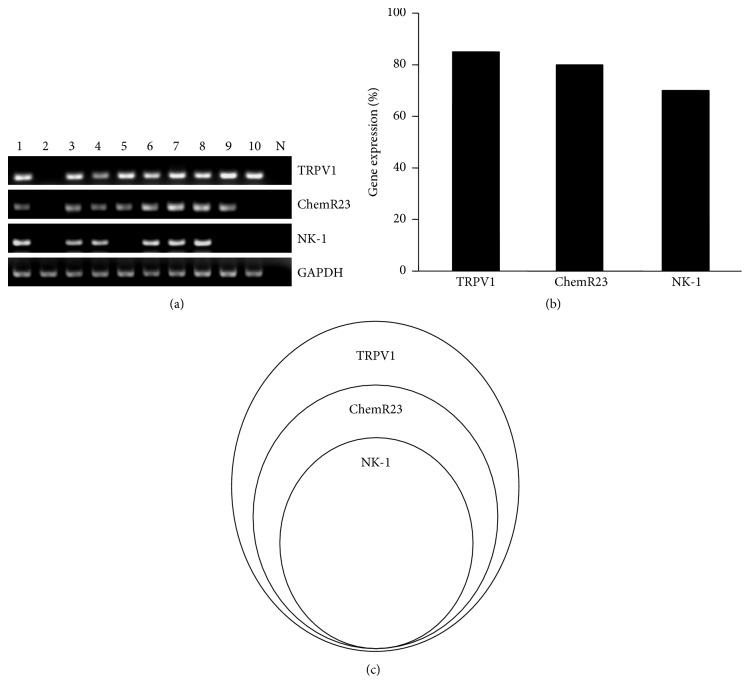
Expression of TRPV1, ChemR23, and NK-1 in a subset of small-sized DRG neurons. (a) Single-cell RT-PCR analysis from 10 individual small-sized DRG neurons showing colocalization of NK-1 with TRPV1 and ChemR23. (b) Expression of TRPV1, ChemR23, and NK-1 by single-cell RT-PCR in dissociated small-sized DRG neurons. (c) A Venn diagram showing the relationship of NK-1^+^, ChemR23^+^, and TRPV1^+^ populations in small-sized dorsal root ganglion (DRG) neurons. Note that all NK-1^+^ neurons also express ChemR23 and TRPV1.

We investigated whether small-sized DRG neurons expressed NK-1, ChemR23, and TRPV1 mRNAs using single-cell RT-PCR. Single-cell RT-PCR analysis conducted selectively in small-sized DRG neurons revealed that approximately 84% (*n* = 42/50), 80% (*n* = 40/50), and 70% (*n* = 35/50) of small neurons expressed TRPV1, ChemR23, and NK-1, respectively (Figures 1(a) and 1(b)). Notably, all NK-1^+^ neurons expressed TRPV1 and ChemR23 (Figure 1(c)).

### 3.2. RvE1 Inhibits the SP-Induced Potentiation of TRVP1

**Figure 2:**
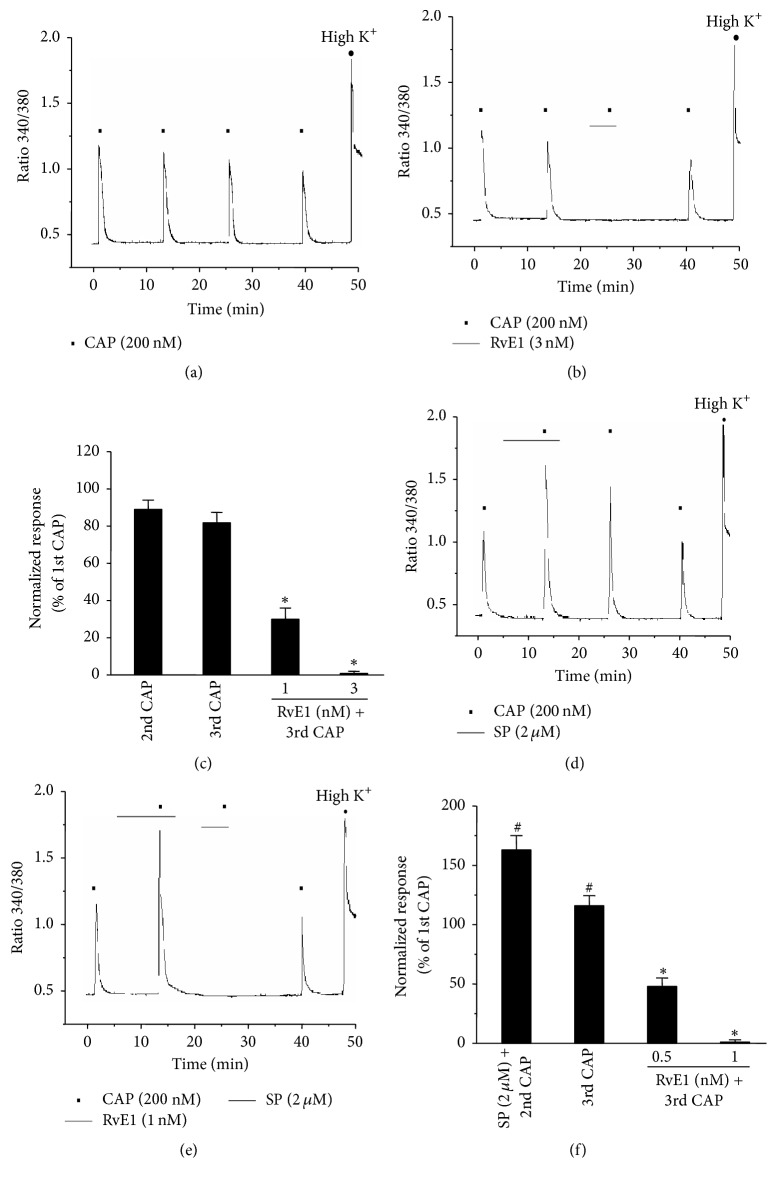
RvE1 inhibits substance P- (SP-) induced potentiation of capsaicin in nociceptive neurons. (a) Capsaicin (CAP, 200 nM) produced consistent [Ca^2+^]i responses without desensitization after repetitive application (intervals of 8~10 min). Cell viability of neurons was confirmed by their response to high K^+^ (50 mM KCl solution) at the end of the experiment. *n* = 18. (b) RvE1 (3 nM) completely inhibited capsaicin-induced [Ca^2+^]i in capsaicin-responsive neurons. Neurons were perfused with RvE1 (3 nM) for 5 min before the third application of capsaicin. *n* = 35. (c) Summary of [Ca^2+^]i responses relative to peak amplitude of first capsaicin responses. Note that RvE1 (1 nM) inhibits 70% of capsaicin-induced [Ca^2+^]i. Results are presented as the mean ± SEM. ^*∗*^ *P* < 0.05; *t*-test versus second capsaicin treatment. (d) Substance P (2 *μ*M) enhanced capsaicin- (200 nM) induced [Ca^2+^]i in small dorsal root ganglion (DRG) neurons (*n* = 29/40). Neurons were perfused with substance P (2 *μ*M) for 10 min before the second application of capsaicin. (e) RvE1 (1 nM, 5 min) completely inhibited substance P-induced potentiation of capsaicin in small DRG neurons. *n* = 20 (f) Summary of [Ca^2+^]i responses relative to peak amplitude of first capsaicin responses. Note that RvE1 (0.5 nM) inhibits 45% of substance P-induced potentiation of capsaicin. Results are presented as the mean ± SEM. ^*∗*^ *P* < 0.05; *t*-test versus second capsaicin treatment. ^#^ *P* < 0.05; *t*-test versus first capsaicin treatment.

Generally, constant capsaicin treatment of nociceptive neurons expressing TRPV1 triggers the Ca^2+^-dependent desensitization of TRPV1 through an intracellular Ca^2+^ influx. In Ca^2+^ imaging experiments using small-sized DRG neurons, 1 mM of external Ca^2+^ solution was used to minimize the desensitization of TRPV1, and the duration of low-concentration capsaicin (200 nM) treatment was kept to a minimum (5 sec) [18, 22]. The cell recovery time (washout) was between 8 and 10 min. When capsaicin was treated four times sequentially, the extreme desensitization of TRPV1 was not observed (Figure 2(a)). Capsaicin treatment of RvE1 (5 min) inhibited TRPV1 in a dose-dependent manner during the third treatment (Figures 2(b) and 2(c)). Furthermore, SP treatment-induced TRPV1 potentiation (10 min treatment, 60% increase) was mediated by the second capsaicin treatment via NK-1 activation, and TRPV1 potentiation (20% increase) was still induced upon the third capsaicin treatment even without SP treatment (Figures 2(d) and 2(f)). However, such a response of TRPV1 potentiation to the third capsaicin treatment was dose-dependently inhibited by perfusion of RvE1 at a notably low concentration (Figures 2(e) and 2(f)).

### 3.3. RvE1 Inhibits SP-Induced Potentiation of TRVP1 via the GPCR Signaling Pathway

**Figure 3:**
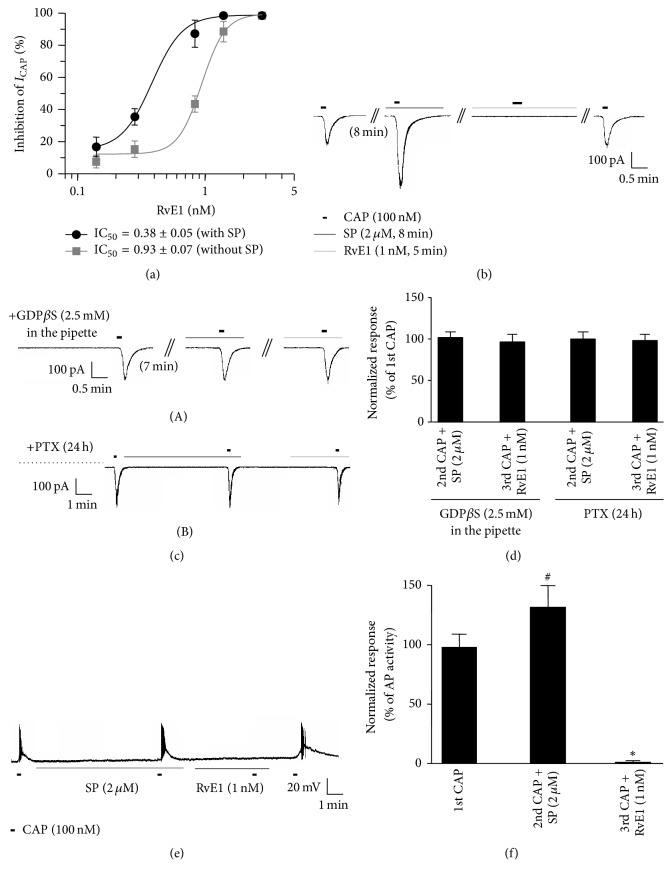
RvE1 inhibits substance P- (SP-) induced potentiation of capsaicin in nociceptive neurons via GPCRs. (a) Dose response curve of RvE1-induced inhibition of TRPV1 currents in small-sized DRG neurons of normal condition (circle, black line) and in SP-treated small-sized DRG neurons (square, gray line). Inset: IC_50_ of TRPV1 current inhibition in normal condition and SP-treated DRG neurons, respectively. (b) RvE1 (1 nM, 5 min) completely inhibited substance P- (2 *μ*M, 8 min) induced potentiation of capsaicin-activated currents in small-sized DRG neurons. *n* = 12. ((c) (A)) Intracellular perfusion of GDP*β*S (2.5 mM, 8 min) blocks both the enhancement and inhibitory effects of capsaicin by SP and RvE1, respectively (*n* = 12). ((c) (B)) Pretreatment of DRG cultures with PTX (0.5 *μ*g/mL, 18–24 h) blocks both the enhancement and inhibitory effects of capsaicin by SP and RvE1, respectively (*n* = 10). (d) Summary of GDP*β*S and PTX blocking the inhibition of SP-induced increases in capsaicin currents and RvE1-mediated inhibition of TRPV1. (e) Current-clamp recording showing that the number of capsaicin-induced action potentials is increased by SP (2 *μ*M, 8 min) and this increase is completely inhibited by RvE1 (1 nM, 5 min). *n* = 10. (f) Summary of the number of action potentials. Results are presented as the mean ± SEM. ^*∗*^ *P* < 0.05; *t*-test versus second capsaicin treatment with SP. ^#^ *P* < 0.05; *t*-test versus third capsaicin treatment with RvE1.

Whole-cell voltage-clamp recording experiments revealed that RvE1 inhibited the capsaicin-induced currents in a dose-dependent manner (IC_50_ = 0.93, IC_50_ = 0.38) under normal conditions without SP treatment and with SP treatment, respectively (Figure 3(a)). TRPV1 further potentiated by SP treatment was completely inhibited by RvE1 even at a lower concentration (Figure 3(b)). We tested whether the SP-induced potentiation of TRPV1 and its inhibition by RvE1 were dependent on the GPCR signaling pathway mediated by NK-1 and Chem23, respectively. When the G*α*i-coupled GPCR inhibitor pertussis toxin and the G-protein inhibitor GDP*β*-S were used, SP-induced TRPV1 potentiation and the inhibitory effect of RvE1 on TRPV1 were both hindered (Figures 3(c) (A and B) and 3(d)). This result indicates that the activation of NK-1 and ChemR23 by SP and RvE1 is governed by the potentiation and inhibition of TRPV1 via a GPCR signaling pathway. Furthermore, the current-clamp recording experiments showed that action potentials induced by capsaicin treatment were further increased following SP treatment (27%), although they were completely inhibited by low concentrations of RvE1 (1 nM). Therefore, our results suggest that RvE1 strongly inhibits TRPV1 activity (Figures 3(e) and 3(f)).

## 4. Discussion

Tissue damage induces inflammatory pain and peripheral inflammation. Generally, inflammatory pain is caused by the peripheral sensitization of primary sensory neurons affected by stimuli from inflammatory mediators [1–3]. The neuropeptide SP is the most commonly known neurotransmitter in pain transmission and is expressed by a subset of unmyelinated nociceptive primary sensory neurons within the DRG [14, 23, 24]. Several electrophysiological studies have suggested that the SP receptor NK-1 is expressed by primary sensory neurons, because SP activates DRG neurons both* in vivo *and* in vitro* [25]. RT-PCR studies have further demonstrated NK-1 mRNAs in cat, rat, and mouse DRG neurons [26, 27]. Specific NK-1 bands were also observed in western blots of DRG neurons, which were further upregulated following complete Freund's adjuvant-induced inflammation [14]. In addition, our single-cell RT-PCR analysis suggested that NK-1 is expressed by approximately 80% of small-sized DRG neurons (Figures 1(a)–1(c)), indicating that peripheral SP associated with tissue damage is involved in the production of inflammatory pain via NK-1 expressed by primary sensory neurons.

TPRV1 is specifically expressed by C-fiber nociceptors and is a critical TRP channel strongly implicated in the genesis of inflammatory pain [28]. Recently, a small-molecule TRPV1 inhibitor was reported to be in the process of development [29]. Interestingly, various lipid mediators function as endogenous inhibitors of TRPV1. Among them, RvE1 is derived from eicosapentaenoic acid demonstrates potent anti-inflammatory and analgesic effects via the suppression of TRPV1 activity in DRG neurons. RvE1 elicits its antinociceptive effects via activation of the G-protein coupled receptor ChemR23, which is widely expressed by neurons (primary sensory neurons and spinal cord neurons), immune cells (macrophages), and microglia [30]. The present study results indicate that ChemR23 is primarily coexpressed with NK-1 and TRPV1 by small-sized DRG neurons (Figure 1(a)), suggesting that TRPV1-positive DRG neurons that coexpress NK-1 are nociceptive neurons involved in inflammatory pain. Furthermore, because ChemR23 is coexpressed in 78% of these TRPV1-positive nociceptors (Figures 1(b) and 1(c)), RvE1 may have an important role in inhibiting inflammatory pain due to TRPV1 activity associated with peripheral inflammation.

Both the peripheral (intraplantar) and central (intrathecal) administration of RvE1 effectively reduce inflammatory pain. Interestingly, RvE1 inhibited TRPV1 in DRG neurons [16]. Similarly, our calcium imaging results indicated that RvE1 acts as a potent endogenous inhibitor of TRPV1 in small-size DRG neurons (Figures 2(b) and 2(c)). SP enhanced-TRPV1 activity induces heat hyperalgesia upon peripheral inflammation [14]. The present calcium imaging experiments confirmed that additional treatment with capsaicin after pretreatment with SP in small-size DRG neurons resulted in capsaicin-mediated increases in TRPV1 potentiation of more than 60% (Figure 2(d)). Most surprisingly, RvE1 completely inhibited SP-potentiated TRPV1 activity at a much lower concentration (1 nM) than that required under normal conditions in the absence of SP (3 nM) (Figures 2(e) and 2(f)). These results indicate that the inflammatory mediator SP plays a role in further potentiating the TRPV1 activity of peripheral neurons and that this activation is inhibited by RvE1 at very low concentrations (1 nM), suggesting that even low concentrations of RvE1 can inhibit the effect of SP on pain induction caused by peripheral tissue damage. Patch clamp results also revealed that RvE1 inhibited capsaicin-induced currents in a dose-dependent manner (IC_50_ = 0.93) under normal conditions in nociceptive neurons (Figure 3(a)). TRPV1 was enhanced by the activity of NK-1 when DRG neurons were treated with SP, and RvE1 in such cases inhibited the capsaicin-induced currents at a concentration half as high (IC_50_ = 0.38) as that required for inhibition under normal conditions (Figure 3(a)). Moreover, RvE1 completely inhibited capsaicin-induced action potentials that had increased by 27% in DRG neurons due to SP (Figures 3(e) and 3(f)). Such inhibition of TRPV1 by RvE1 in DRG neurons is known to be mediated by inhibitory signaling of the G-protein coupled receptor ChemR23 [16, 18]. Pretreatment of DRG neurons with pertussis toxin for 18 h and GDP*β*-S resulted in the inhibition of RvE1 with respect to the SP-induced potentiation of TRPV1 (Figures 3(a) and 3(b)). These data suggest that RvE1 inhibits SP-induced TRPV1 potentiation via an inhibitory signaling pathway activated by specific PTX-sensitive/G*α*i-coupled GPCRs. It is surprising that a very low concentration of RvE1 inhibits TRPV1 activity that has been enhanced by SP treatment. In such cases, it can be presumed that RvE1 can inhibit TRPV1 responses more strongly at lower concentrations because the ChemR23 gene becomes overexpressed in nociceptor neurons due to the presence of inflammatory substances, including SP. Indeed, ChemR23 mRNA levels were increased by various inflammatory substances in human monocytes, macrophages, and cornea where ChemR23 receptors are expressed [31, 32]. The differential expression patterns of ChemR23 on stimulated SP and the possible inhibitory effect of RvE1 on SP-induced TRPV1 potentiation in small DRG neurons may indicate that the role of ChemR23 signaling differs depending on the presence/absence of SP. Further studies regarding the cross-activity of NK-1 activation by SP on both the potentiation of TRPV1 and overexpression of ChemR23 via a GPCR signaling pathway in heterologous HEK293 cells overexpressing NK-1, TRPV1, and ChemR23 are therefore required. The results of the present study suggest the therapeutic potential of RvE1 as an endogenous inhibitor of inflammatory pain, because only a very low concentration of RvE1 was required to inhibit TRPV1 activity enhanced by peripheral SP in nociceptive neurons.

In summary, the present study indicates that the nociceptor TRPV1, the SP receptor NK-1, and the RvE1 receptor ChemR23 are coexpressed by small-sized DRG neurons. Furthermore, SP potentiates TRPV1 activity in nociceptive neurons that is completely inhibited by RvE1 at very low concentrations (1 nM), and this inhibition occurs via a GPCR signaling pathway. Therefore, we propose that RvE1 can effectively inhibit SP-induced TRPV1 potentiation due to peripheral inflammation.

## Figures and Tables

**Table 1:** List of DNA primer sequences designed for single-cell RT-PCR.

Target gene (product length)^a^		Outer primers	Inner primers	Genbank number

TRPV1 (273 bp, 203 bp)	Forward	TGATCATCTTCACCACGGCTG	AAGGCTTGCCCCCCTATAA	NM_001001445.1
	Reverse	CCTTGCGATGGCTGAAGTACA	CACCAGCATGAACAGTGACTGT	

ChemR23 (481 bp, 273 bp)	Forward	GCCTCGCTAAAGCAACAAAC	TGGAGGAGTTCCACAAACAC	NM_027852.2
	Reverse	GCCAGCCTGTGCTATCTTAAT	GAGGCCCTTGCTTCAGAAT	

NK1 (401 bp, 204 bp)	Forward	TCAGCCCTGGGAACCTATAA	CCCTGCTCCAAAGACCATTTA	NM_009313.5
	Reverse	GCTCCTCTCTAAGCAAGAACAG	CCACAGGTTGGAGGGAATG	

GAPDH (367 bp, 313 bp)	Forward	AGCCTCGTCCCGTAGACAAAA	TGAAGGTCGGTGTGAACGAATT	XM_001473623.1
	Reverse	TTTTGGCTCCACCCCTTCA	GCTTTCTCCATGGTGGTGAAGA	

(n1, n2)^a^: n1 and n2 indicate the sizes of PCR products obtained from the outer and inner primers, respectively.

## Abbreviations

SP: Substance P
NK: Neurokinin
TRPV1: Transient receptor potential vanilloid subtype 1
RvE1: Resolvin E1
GPCR: G-protein coupled receptor
DRG: Gorsal root ganglion
RT-PCR: Reverse-transcription polymerase chain reaction.
